# Structural Covariance of the Ipsilesional Primary Motor Cortex in Subcortical Stroke Patients with Motor Deficits

**DOI:** 10.1155/2022/1460326

**Published:** 2022-03-10

**Authors:** Xinyuan Chen, Mengcheng Li, Naping Chen, Huimin Lai, Ziqiang Huang, Yuqing Tu, Qunlin Chen, Jianping Hu

**Affiliations:** ^1^Department of Rehabilitation, The First Affiliated Hospital of Fujian Medical University, Fuzhou, Fujian 350005, China; ^2^Department of Radiology, The First Affiliated Hospital of Fujian Medical University, Fuzhou, Fujian 350005, China

## Abstract

The analysis of structural covariance has emerged as a powerful tool to explore the morphometric correlations among broadly distributed brain regions. However, little is known about the interactions between the damaged primary motor cortex (M1) and other brain regions in stroke patients with motor deficits. This study is aimed at investigating the structural covariance pattern of the ipsilesional M1 in chronic subcortical stroke patients with motor deficits. High-resolution T1-weighted brain images were acquired from 58 chronic subcortical stroke patients with motor deficits (29 with left-sided lesions and 29 with right-sided lesions) and 50 healthy controls. Structural covariance patterns were identified by a seed-based structural covariance method based on gray matter (GM) volume. Group comparisons between stroke patients (left-sided or right-sided groups) and healthy controls were determined by a permutation test. The association between alterations in the regional GM volume and motor recovery after stroke was investigated by a multivariate regression approach. Structural covariance analysis revealed an extensive increase in the structural interactions between the ipsilesional M1 and other brain regions in stroke patients, involving not only motor-related brain regions but also non-motor-related brain regions. We also identified a slightly different pattern of structural covariance between the left-sided stroke group and the right-sided stroke group, thus indicating a lesion-side effect of cortical reorganization after stroke. Moreover, alterations in the GM volume of structural covariance brain regions were significantly correlated to the motor function scores in stroke patients. These findings indicated that the structural covariance patterns of the ipsilesional M1 in chronic subcortical stroke patients were induced by motor-related plasticity. Our findings may help us to better understand the neurobiological mechanisms of motor impairment and recovery in patients with subcortical stroke from different perspectives.

## 1. Introduction

Globally, stroke is the main cause of acquired adult disability. Motor deficits are the most common symptom after stroke and can be caused by ischemic or hemorrhagic damage to the motor pathway [1]. Motor recovery may spontaneously occur during the first few months even in stroke patients who have never received rehabilitation therapy [2]. The neural mechanisms underlying the recovery of motor function after stroke are complex and sometimes confusing; these mechanisms have been associated with functional and structural reorganization within the brain.

Numerous neuroimaging techniques have been extensively used to investigate the alterations in brain function and structure associated with motor recovery after stroke [3]. Many neuroimaging studies have demonstrated that stroke-induced brain structural plasticity occurs not only at the perilesional regions but also at the distant cortical regions remote from the lesion. For instance, structural magnetic resonance imaging- (MRI-) based morphometric studies have revealed that the recovery of motor function after focal damage to the motor pathway was accompanied by profound plastic alteration in cortical morphology, including the sensorimotor cortex, basal ganglia, cerebellum, and cognitive-related cortical regions [4–8]. However, these studies mostly focused on plastic alterations in the brain regions after stroke; the interactive relationships between different regions of the brain have yet to be investigated in detail. Recently, structural covariance analysis has emerged as a powerful tool with which to explore the morphometric correlations among broadly distributed brain regions [9, 10]. Structural covariance can be evaluated by various metrics, such as gray matter volumes, cortical thickness, and cortical gyrification [10]. Compared with functional MRI (fMRI) and diffusion MRI networks that are routinely constructed from interregional association or connectivity for an individual image, structural covariance networks are constructed from interregional correlations based on a group of individual images [11]. Recent studies have highlighted that structural covariance could partially reflect anatomical connectivity between cortical regions; these studies have proved that this methodology has topological properties that are similar to a functional brain network [12–14]. Although its biological implication remains controversial, structural covariance is thought to arise from experience-related plasticity or mutually trophic influences [15]. Therefore, the analysis of structural covariance may provide complementary information to fMRI and diffusion tensor imaging (DTI) networks and help to further clarify the specific mechanisms that underlie neurological and psychiatric disorders. Thus far, structural covariance analysis has successfully been used to explore changes in the anatomical coupling of healthy subjects and patients with various psychiatric and neurological disorders, such as schizophrenia, major depressive disorder, Parkinson's disease, and Alzheimer's disease [14, 16–22].

A limited number of studies have investigated alterations in the structural covariance network after stroke. For example, Abela and coworkers demonstrated that structural covariance alterations in the thalamocortical network were associated with motor recovery after ischemic stroke by using principal component analysis based on tensor-based morphometry [23]. In another study, Wang and colleagues used brain volumetry from multi-atlas-based anatomical segmentation and found that chronic stroke patients with internal capsule infarct showed extensive changes of structural covariance involving a network at the whole-brain level and anatomical reorganization patterns that were dependent on the side of the lesion [24]. Another recent study used a seed-based structural covariance approach to detect progressive gray matter atrophy in the bilateral cerebellar cortex and abnormal structural covariance patterns in patients with pontine infarction [25]. Collectively, these studies applied different structural covariant models and revealed the interaction between damaged brain regions and other distant brain functional regions after stroke from a variety of different perspectives.

The extent of damage to motor-related brain regions has been closely related to motor recovery in patients suffering from stroke [26, 27]. Structural covariance measurements can be performed by a range of different approaches, including seed-based analysis, principal component analysis, or graph analysis [11]. Seed-based structural covariance analysis measures interactions between the seed region and the remaining brain regions and can help to investigate the structural plasticity induced by damage in the brain tissue. However, little is known about the structural covariance alterations induced by damage to the primary motor cortex (M1) in subcortical stroke. Therefore, in the present study, we aimed to investigate covariant alterations between the ipsilesional M1 and other brain regions in patients suffering from chronic subcortical stroke by applying seed-based structural covariance analysis. We hypothesized that structural covariance alterations of the ipsilesional M1 are closely related to motor recovery in patients with chronic subcortical stroke. Furthermore, in view of cerebral hemispheric asymmetry, a lesion-side effect has been demonstrated in previous studies [6, 7, 24]; we further hypothesized that stroke patients with lesions in different hemispheres will exhibit different patterns of structural covariance.

## 2. Materials and Methods

### 2.1. Participants

Fifty-eight chronic subcortical stroke patients with motor deficits (29 with left-sided lesions and 29 with right-sided lesions) were recruited from the First Affiliated Hospital of Fujian Medical University (Fuzhou, China). The inclusion criteria were as follows: (1) first-onset unilateral stroke (ischemic or hemorrhagic stroke), (2) a single subcortical lesion and no previous cerebrovascular events, (3) the time after stroke onset was >6 months, (4) no history of neurosurgery, (5) no history of other neuropsychiatric diagnoses, and (6) no contraindications for MRI scanning. Motor impairments were evaluated using the Fugl-Meyer assessment for upper extremity (FMA-UE) on the same day as MRI scan acquisition. The FMA-UE score ranges from 0 to 66; the higher the FMA-UE score, the better the performance. Fifty age-matched healthy subjects were also recruited as healthy controls (HC). The study was approved by the local Medical Research Ethics Committee, and all participants signed informed consent.

### 2.2. MR Imaging Data Acquisition

All MRI scans were performed with a 3-Tesla Siemens Skyra scanner with a 20-channel head-neck coil. Three-dimensional sagittal T1-weighted images were acquired using T1-weighted 3D magnetization-prepared rapid gradient-echo (MPRAGE) sequences with the following parameters: repetition time = 2300 ms, echo time = 2.3 ms, inversion time = 900 ms, flip angle = 8, field of view = 256 × 256 mm^2^, matrix = 256 × 256, bandwidth of 200 Hz/Px, voxel size = 1.0 × 1.0 × 1.0 mm, a total of 192 slices, and acquisition time = 5.18 min.

### 2.3. Lesion Mapping

**Figure 1:**
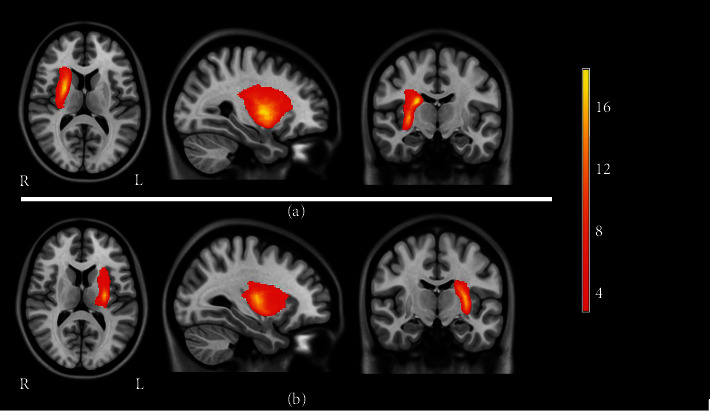
Lesion probability map for patients with subcortical stroke. (a) Lesion distribution in right-sided stroke patients; (b) lesion distribution in left-sided stroke patients. Color bar denotes lesion incidence frequency. Abbreviations: L = left hemisphere; R = right hemisphere.

The location of the lesion in each patient was manually delineated to create a lesion mask on 3D T1-weighted MRI images by an experienced neuroradiologist using MRIcron software (http://www.mccauslandcenter.sc.edu/mricro/mricron/). The T1-weighted images and lesion masks for all stroke patients were then spatially normalized to the Montreal Neurological Institute (MNI) space. The probability maps of lesion distribution (left and right side) were created by overlapping the normalized lesion mask on the MNI template (see Figure 1).

### 2.4. Image Preprocessing

All of the 3D T1 structural MRI data were processed using the VBM8 toolbox implemented in Statistical Parametric Mapping software (SPM 8; The Wellcome Department of Imaging Neuroscience, London, UK; http://www.fil.ion.ucl.ac.uk/spm), using the following steps: (1) all MRI images were visually inspected and manually reoriented to the anterior-posterior commissural plane by an experienced neuroradiologist; (2) whole-brain MR images were segmented into gray matter (GM), white matter (WM), and cerebrospinal fluid (CSF); (3) the segmented images were spatially normalized to the MNI space using Diffeomorphic Anatomical Registration Through Exponentiated Lie Algebra (DARTEL) normalization; (4) the normalized GM images were then modulated to obtain volumetric information and then smoothened with an 8 mm full width at half-maximum (FWHM) Gaussian kernel for structural covariance analysis.

### 2.5. Group Comparison of GM Volume

A voxel-wise two-sample *t*-test, based on the smoothened and normalized GM images, was conducted to identify differences between stroke patients (the left-sided or right-sided groups) and healthy controls (HC) in SPM8. The gender, age, and total intracranial volume (TIV, the sum of GM, WM, and CSF) for each participant were entered as covariates of no interest. Results were considered significant with cluster-level *p* values < 0.05; family-wise error (FWE) correction was performed where clusters formed at *p* < 0.05 FWE corrected.

### 2.6. Structural Covariance Analysis

Seed-based structural covariance analysis was conducted using the Brain Covariance Connectivity Toolkit (BCCT) based on the MATLAB platform [28]. A mask of the primary motor cortex (M1) was firstly created using Brodmann area (BA) 4 defined by the BA atlas available in MRIcron software. Then, we constructed a spherical seed ROI with a radius of 4.0 mm and centered MNI coordinates at the peak voxel within the significant cluster of the ipsilesional M1 in each stroke group relative to the HC group. Partial correlations across subjects were explored between the ipsilesional seed ROIs and other regions throughout the whole brain using Pearson's correlation coefficients for each group while controlling the gender, age, and total intracranial volume (TIV) as confounding covariates. The resulting correlation maps were observed at a threshold of *p* < 0.001. Finally, a total of four structural covariance maps were obtained: two structural covariance maps of the ipsilesional seed ROI for patients with left-sided lesions and the HC group,and two other structural covariance maps for patients with right-sided lesions and the HC group.

In order to further investigate the difference in structural covariance alterations between each stroke group and the HC group, a nonparametric permutation test was used to test for the statistical significance of the between-group differences. We performed permutation tests 5000 times by randomly rearranging and regrouping the two groups of participants and recorded all of the differences between the two groups; these were used to create the null distribution. The significance of the true difference between two groups was calculated by using the normal distribution function according to the null distribution [28–30]. The comparison results were set at *p* < 0.001 uncorrected, and the differences in correlation coefficients between the two groups (the stroke group minus the HC group) at the positive brain regions were reported.

### 2.7. The Association between Clinical Assessments and Alterations in GM Volume

To investigate the association between structural covariance alterations and motor impairment, we extracted the mean GM volumes within the seed regions and significant clusters within the structural covariance analysis. In line with previous research [22], we then used a multivariate regression model to explore the association between GM volume alterations within these regions and the motor function for each stroke group using R software (R 3.4, https://www.R-project.org/). In this analysis, the FMA-UE scores were defined as the dependent variable, while age, gender, stroke type (ischemic or hemorrhagic stroke), lesion size, and disease duration were entered as covariates since they were potentially confounding variables for motor recovery after stroke. Furthermore, the GM volume in each region was separately entered as the independent variable on top of the covariates in the linear regression model on each occasion. The significance level of the fitted model and the regression coefficient of the explained variable before and after adding the GM volume of this region were then assessed. The significance level of the regression coefficient for the explained variable was used to indicate the association between GM volume alterations in this region and the FMA-UE scores.

## 3. Results

### 3.1. Clinical and Behavioral Data

The demographic and clinical data of participants are summarized in Table 1. There were no significant differences in terms of either age or gender when comparing the left-sided stroke patients, the right-sided stroke patients, and the healthy controls. The lesion size, stroke duration, stroke type, and FMA-UE scores were not significantly different when compared between the left-sided and right-sided stroke groups.

### 3.2. GM Volume Differences between Groups

**Figure 2:**
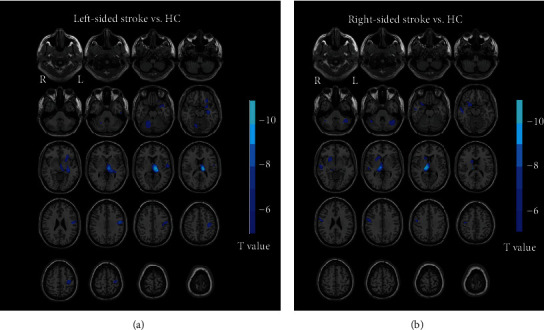
GM volume alterations in stroke patients. (a) Group comparison between left-sided stroke patients and healthy controls (*p* < 0.05, FWE corrected). (b) Group comparison between right-sided stroke patients and healthy controls (*p* < 0.05, FWE corrected). Abbreviations: L = left hemisphere; R = right hemisphere; HC = healthy controls.

Compared with the HC group, the stroke groups (left-sided stroke and right-sided stroke) showed a reduced GM volume in the ipsilesional precentral gyrus, postcentral gyrus, thalamus, putamen, insula, inferior frontal gyrus, parahippocampal gyrus, and the contralesional cerebellum (see Figure 2). Further details relating to these regions are provided in Table 2. There was no significant increase in GM volume in the stroke groups (right-sided stroke and left sided stroke) when compared with the HC group.

### 3.3. Seed-Based Structural Covariance Analysis

The peak MNI coordinates of the ipsilesional M1 were *x* = −34.5, *y* = −27, and *z* = 61.5 for the left-sided stroke group and *x* = 42, *y* = −16.5, and *z* = 48 for the right-sided stroke group; these were identified as the seed region for seed-based structural covariance analysis.

**Figure 3:**
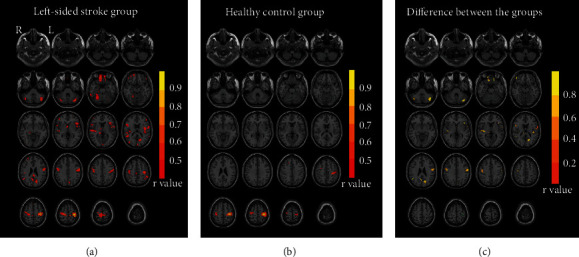
Structural covariance maps and group comparisons in left-sided stroke patients and healthy controls. (a) Structural covariance map of the ipsilesional M1 (left side) in the left-sided stroke group (*p* < 0.001, uncorrected). (b) Structural covariance map of the ipsilesional M1 (left side) in the healthy controls (*p* < 0.001, uncorrected). (c) Group comparison between the left-sided stroke group and the healthy controls group (*p* < 0.001, uncorrected) and the differences in correlation coefficients between the two groups (stroke group minus healthy controls group) in the positive brain regions. The cyan circle shows the location of the seed point (the ipsilesional M1). Abbreviations: L = left hemisphere; R = right hemisphere; HC = healthy controls.

The seed-based group correlation maps and group comparison of the left-sided stroke group and the HC group are shown in Figure 3 and Table 3. Compared with the healthy controls, the left-sided stroke patients showed increased correlation strength in the bilateral precentral gyrus, bilateral prefrontal cortex (Frontal_Sup_Orb_L, Frontal_Inf_Tri_L, Rolandic_Oper_L, Frontal_Inf_Orb_R, Frontal_Inf_Oper_R, Frontal_Sup_Orb_R), bilateral insula, bilateral parieto-occipital cortex (Cuneus_L, Occipital_Mid_R, Precuneus_L, ), and bilateral cerebellum (Cerebelum_Crus1_L, Cerebelum_Crus1_R), Angular_R, and Thalamus_R.

**Figure 4:**
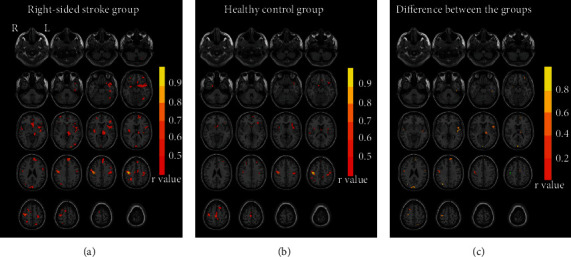
Structural covariance maps and group comparisons in the right-sided stroke patients and healthy controls. (a) Structural covariance map of the ipsilesional M1 (right side) in the right-sided stroke group (*p* < 0.001, uncorrected). (b) Structural covariance map of the ipsilesional M1 (right side) in the healthy controls (*p* < 0.001, uncorrected). (c) Group comparison between the right-sided stroke group and the healthy controls (*p* < 0.001, uncorrected) and the differences in correlation coefficients between the two groups (stroke group minus healthy controls) in the positive brain regions. The cyan circle shows the location of the seed point (the ipsilesional M1). Abbreviations: L = left hemisphere; R = right hemisphere; HC = healthy controls.

The seed-based group correlation maps and group comparison of the right-sided stroke group and the HC group are shown in Figure 4 and Table 3. Compared with the healthy controls, the right-sided stroke patients showed increased correlation strength in the bilateral precentral gyrus, bilateral prefrontal cortex (Frontal_Mid_L, Frontal_Sup_Medial_L, Frontal_Sup_R, Rolandic_Oper_L, Frontal_Mid_Orb_L, Supp_Motor_Area_L), bilateral parieto-occipital cortex (Cuneus_L+R, Parietal_Sup_L, Occipital_Mid_L, Occipital_Sup_R), and bilateral temporal lobe (Temporal_Inf_L, Temporal_Sup_R), Amygdala_R, and Thalamus_L.

Relative to healthy controls, there was no reduction in the structural covariance regions in the left-sided stroke patients or the right-sided stroke patients.

### 3.4. The Association between Clinical Assessments and Alterations in GM Volume

**Figure 5:**
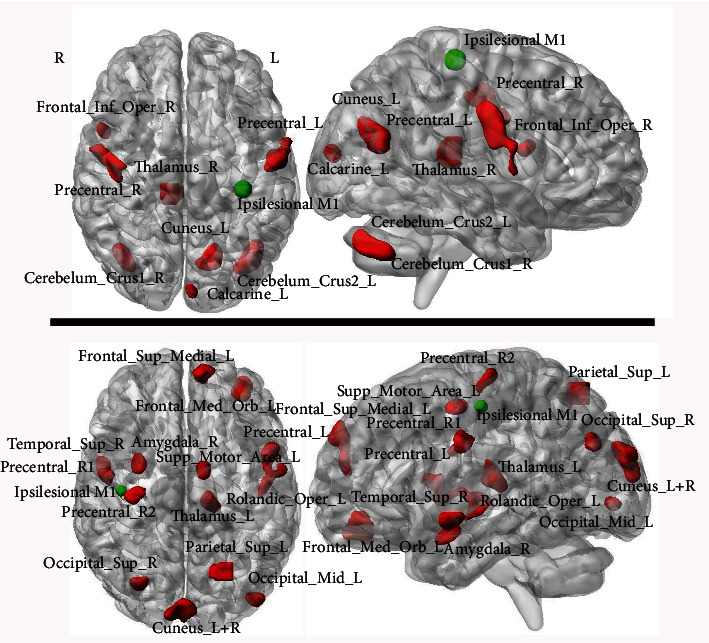
The association between FMA-UE scores and GM volume alterations. (a) Brain regions showing significant correlations with FMA-UE scores in the left-sided stroke group. (b) Brain regions showing significant correlations with FMA-UE scores in the right-sided stroke group. The cyan circle is the location of the seed point (the ipsilesional M1). Abbreviations: L = left hemisphere; R = right hemisphere.

The results of the multivariate regression model are shown in Figure 5 and Tables 4 and 5. After adjusting for age, gender, stroke type, lesion size, and disease duration, several brain regions in the left-sided stroke patients, including the seed region, Thalamus_R, Frontal_Inf_Oper_R, Cuneus_L, Calcarine_L, the bilateral precentral gyrus, and the bilateral cerebellum, were positively associated with FMA-UE scores. In contrast, most brain regions in the right-sided stroke patients were positively associated with the FMA-UE scores except for Temporal_Inf_L, Frontal_Mid_L, and Frontal_Sup_R.

The table shows the results of the multivariate regression model. The variables were added to the regression model in a stepwise fashion. ^a^*R*^2^ represents the goodness-of-fit of the regression model; ^b^the significance level of the regression model; ^c^unstandardized coefficient (beta) with SE (standard error) of the explained variable; ^d^the significance level of the regression coefficient of the explained variable; ^e^the lesion size was normalized by TIV (% of TIV); ^f^duration: disease duration; ^g^TIV: total intracranial volume; ^h^ipsilesional M1: the seed region; ^i^the significant variables in the regression model and regression coefficient.

## 4. Discussion

In the present study, we analyzed structural covariance alterations between the ipsilesional M1 and other brain regions in patients with chronic subcortical stroke. Structural covariance analysis revealed an extensive increase in the structural interactions between the ipsilesional M1 and other brain regions in stroke patients, involving not only motor-related brain regions but also non-motor-related brain regions. Moreover, the GM volume alterations in some of the brain regions were significantly correlated to motor function scores in stroke patients.

In our study, two groups of stroke patients (the left-sided lesion group and the right-sided lesion group) demonstrated a similar pattern of brain atrophy involving the ipsilesional sensorimotor cortex, the ipsilesional cortex related to the limbic system, and the contralesional cerebellum. Among these regions of atrophy, the sensorimotor cortex and the limbic brain regions such as hippocampus, amygdala, and insular cortex were densely connected with subcortical structures (for example, the thalamus) [8, 31]. The reduction of GM volume in these regions could be caused by axonal degeneration secondary to subcortical damage. In addition, the GM volumetric loss in the contralesional cerebellum may result from crossed cerebellar diaschisis in the cortico-cerebellar loop due to damage in the supratentorial structure [4, 32]. Moreover, the topographic similarity in terms of brain atrophy and lesion location between the left-sided stroke group and the right-sided stroke group suggested a similar degree of injury in the two groups. This also helped to clarify the potentially relevant mechanisms underlying the lesion-side effect after subcortical stroke.

Subcortical stroke usually affects the subcortical motor pathway, resulting in secondary cortical impairment and functional reorganization. Although the underlying mechanisms remain unclear, the structural damage of the ipsilesional M1 has been considered to be closely associated with motor recovery after stroke [7, 33, 34]. In the present study, seed-based structural covariance analysis revealed multiple brain regions, including the perilesional regions and the remote brain regions within the contralesional hemisphere, thus demonstrating increased correlation strength of the gray matter volume with the ipsilesional M1. These regions not only involved the cortex that showed significant atrophy relative to the healthy controls but also the cortex, which presented no significant volumetric difference from the healthy controls. This suggests that the structural remodeling change caused by the interaction between the ipsilesional M1 and other brain regions in patients with chronic stroke is a whole brain network-specific process that is independent of the lesion side and lesion location. These alterations of structural covariance were also consistent with the concept of connectional diaschisis that is defined as changes in the structural and functional connectivity among brain regions distant to the lesion [35, 36].

The interaction between the two hemispheres in the motor network is thought to play an important role in motor recovery after stroke. Functional reorganizational processes may occur on the ipsilesional and contralesional hemispheres, involving the disinhibition of redundant neural circuits, the recruitment of functionally homologous regions, and the formation of new neural connections to take over the functions of the damaged neurons [34]. For example, several studies demonstrated that increased ipsilesional hemispheric recruitment appeared to be associated with greater motor gains while increased contralesional M1 recruitment seemed to be associated with poorer motor recovery [37–39]. The reestablishment of normalized hemispheric balance between the two sensorimotor areas has been commonly demonstrated in well-recovered patients [40]. Our results also identified that motor-related regions, such as the bilateral sensorimotor cortex and cerebellum, showed strengthened correlations with the ipsilesional M1 relative to the healthy controls. Moreover, these motor-related regions with strengthened correlations did not fully overlap with the motor covariant network in the HC group, thus suggesting drift and the reshaping of the motor covariant network after stroke. However, the current study focused on interregional correlations in the morphological properties based on a group of subjects. In future studies, the grouping of stroke patients based on prognosis (for example, complete recovery versus partial recovery) may be helpful to further clarify the role of the structural covariance patterns of motor networks in motor recovery after stroke.

In the present study, some cognitive-related brain regions, such as the insular, orbital frontal cortex, middle frontal gyrus, inferior frontal gyrus, medial cingulate cortex, precuneus and amygdala, exhibited strengthened correlations with the ipsilesional M1 in both groups of patients. The structural plasticity of these cognitive-related brain regions has been extensively reported to play an important role in motor recovery after stroke [8, 25, 41, 42]. Moreover, neurocognitive therapy has been demonstrated to have beneficial effects on motor recovery in stroke patients [43]. Therefore, we speculated that the synchronous gray matter changes between motor and cognitive-related brain regions may assist the recruitment of cognitive resources during motor recovery after stroke and might compensate for the impaired anatomical connections caused by stroke, at least to some extent.

The lesion-side effect of cortical reorganization after stroke has been described by previous studies [6, 24]. In addition to motor deficit, left-sided dominant stroke patients usually present with impaired language function, while right-sided dominant stroke patients usually present with unilateral spatial neglect [44]. Our present results also revealed a slightly different pattern of structural covariance for different-sided stroke patients. The left-sided stroke group showed strengthened correlations in language-related regions (the pars opercularis and pars triangularis of the inferior frontal gyrus, BA44/45),while the right-sided stroke group did not. These regions belong to Broca's regions, which are considered to be a motor speech-production area, involving motor action understanding and imitation [45]. Interaction between Broca's region and the primary motor cortex plays an important role in motor and language function recovery after stroke [46, 47]. In contrast, in the right-sided stroke group, we observed more extensive structural covariance in the bilateral superior posterior parietal cortex and the ipsilesional ventral premotor cortex relative to the left-sided stroke group. As key parts of the parietofrontal network, alterations in the functional and structural connectivity between these brain regions and the ipsilesional M1 have been closely associated with motor deficiency and spatial neglect in stroke patients with right hemisphere lesion [48]. Therefore, we speculated that the different covariance patterns between left-sided stroke and right-sided stroke might be influenced by the lateralization of human brain function.

Finally, we evaluated the association between motor recovery and the alteration of GM volume in the ipsilesional M1 and significant regions of structure covariance. After controlling for confounding factors, the GM alteration of motor-related or cognitive-related brain regions showed significant positive association with the FMA-UE scores in both stroke groups. Several recent studies have demonstrated that interregional interactions in terms of morphological properties might primarily reflect the synchronous effects on the connected regions induced by common experience-related plasticity and mutual neurotrophic influence [25, 49]. Thus, the structure covariance pattern of the ipsilesional M1 might reflect motor-related structural plasticity in chronic subcortical stroke patients.

Several limitations should be noted in the current study. First, although we restricted our study cohort to stroke patients with subcortical lesions, there was significant heterogeneity in lesion size and location across different patients. Second, the present study included different types of stroke. In order to reduce this heterogeneity, we only included chronic stroke patients and kept the proportion of stroke types consistent for each group. Third, the current study was a cross-sectional study. In the future, a longitudinal study with a larger sample size may provide more information about the dynamic alterations of structural covariance patterns after stroke; such research is important if we are to fully investigate the biological basis of structural covariance.

## 5. Conclusions

In conclusion, this study described patterns of structural covariance in the ipsilesional M1 of chronic subcortical stroke patients. Our results also revealed the lesion-side effect of structural covariance patterns for the ipsilesional M1 in stroke patients with lesions in different hemispheres. The association between FMA-UE scores and the GM volume of structural covariance brain regions supported the fact that the structural covariance patterns of the ipsilesional M1 were induced by motor-related plasticity. These findings may help us to better understand the neurobiological mechanisms of motor impairment and recovery in subcortical stroke patients from different perspectives.

## Figures and Tables

**Table 1:** Demographic and clinical data of stroke patients and healthy controls.

Characteristics	HC (*n* = 50)	Left (*n* = 29)	Right (*n* = 29)	*p*-value
Age (years)	52.06 ± 11.36	56.79 ± 12.18	56.4 ± 9.36	0.11
Sex (male/female)	22/28	11/18	11/18	0.82
Type (I/H)		12/17	12/17	1.0
Duration (months)		8.78 ± 3.04	8.06 ± 3.04	0.51
Lesion size (ml)		4.88 ± 5.26	5.09 ± 5.25	0.86
Fugl-Meyer assessment				
FMA-UE		33.45 ± 19.52	36.48 ± 21.71	0.58

Stroke type: I ischemic; H hemorrhagic; FMA-UE: Fugl-Meyer assessment for upper extremity. A two-sample *t*-test was used to compare groups for continuous variables whereas the chi-squared test was used to compare groups for categorical variables; statistical analysis was carried out with R software (R 3.4).

**Table 2:** Gray matter volume (GMV) differences between stroke patients and healthy controls (*p* < 0.05, FWE corrected).

Brain regions and percentage of cluster (%)^a^	Brodmann area (BA)	Cluster (voxels)	Peak MNI coordinate	Peak *T* value
Left-sided stroke patients				
Precentral_L (49.35) Postcentral_L (34.40) Rolandic_Oper_L (7.4)	BA4/6	3218	-51 -9 33	9.17
Thalamus_L (40.76) Hippocampus_L (13.18)		5407	-13.5 -21 13.5	12.13
Frontal_Inf_Orb_L (22.92) Insula_L (16.61) Temporal_Pole_Sup_L (15.11) Frontal_Mid_Orb_L (12.61) Putamen_L (11.20)	BA11/47	2101	-27 27 -10.5	6.99
Temporal_Inf_L (90.23)	BA20	256	-49.5 -31.5 -19.5	6.31
Cerebelum_6_R (74.79) Cerebelum_4_5_R (12.26)		1745	15 -52.5 -25.5	6.61
Right-sided stroke patients				
Precentral_R (39.10) Postcentral_R (53.63)	BA4/6	847	52.5 -9 28.5	6.49
Temporal_Sup_R (52.33) Temporal_Mid_R (46.58)	BA21	959	58.5 -10.5 -13.5	6.44
Thalamus_R (39.96) Caudate_R (9.50) Putamen_R (9.28)		4956	12 -21 13.5	11.91
Parahippocampal_R (72.27)		119	25.5 -18 -28.5	5.87
Cerebelum_Crus1_L (69.60) Cerebelum_6_L (15.20)		1141	-39 -57 -31.5	6.48
Cerebelum_Crus1_L (93.75)		299	43.5 -55.5 -30	5.65

^a^Percentage overlap of cluster with the automated anatomical labeling (AAL) atlas (only >5% are reported).

**Table 3:** Regions showing significant differences in structural covariance when compared between stroke patients and healthy controls.

Brain regions	Brodmann area (BA)	Peak MNI coordinate	Difference between groups^a^	Cluster (voxels)	*p* value
Left-sided stroke patients					
Precentral_L	BA4/6	-61.5 -4.5 30	0.78	961	<0.001
Precentral_R	BA4/6	43.5 -21 42	0.65	627	<0.001
Cuneus_L	BA31	-18 -67.5 15	0.76	650	<0.001
Insula_R	BA13	40.5 -16.5 9	0.82	383	<0.001
Frontal_Inf_Tri_L	BA45	-39 24 6	0.68	222	<0.001
Angular_R		46.5 -55.5 28.5	0.88	150	<0.001
Insula_L	BA13	-37.5 -13.5 21	0.60	115	<0.001
Thalamus_R		10.5 -27 15	0.65	67	<0.001
Frontal_Inf_Orb_R		51 46.5 -12	0.80	85	<0.001
Calcarine_L	BA18	-4.5 -90 15	0.60	67	<0.001
Rolandic_Oper_L	BA14	-40.5 -24 15	0.65	100	<0.001
Frontal_Inf_Oper_R	BA44	52.5 7.5 15	0.60	97	<0.001
Frontal_Sup_Orb_R	BA11	21 48 -22.5	0.74	67	<0.001
Precuneus_R	BA31	10.5 -52.5 24	0.74	61	<0.001
Frontal_Sup_Orb_L		-15 30 -24	0.77	58	<0.001
Occipital_Mid_R		37.5 -79.5 13.5	0.73	52	<0.001
Cerebelum_Crus1_R (aal)		36 -75 -36	0.72	132	<0.001
Cerebelum_Crus1_L (aal)		-43.5 -69 -39	0.85	722	<0.001
Right-sided stroke patients					
Rolandic_Oper_L	BA22/24	-48 -15 1.5	0.74	708	<0.001
Cuneus_L+R	BA18/19	1.5 -91.5 27	0.79	607	<0.001
Precentral_R1^b^	BA4/6	49.5 -3 25.5	0.50	504	<0.001
Frontal_Sup_Medial_L	BA9	-13.5 60 33	0.67	355	<0.001
Thalamus_L		-16.5 -22.5 12	0.64	318	<0.001
Temporal_Inf_L	BA20	-43.5 -24 -27	0.67	269	<0.001
Occipital_Sup_R		25.5 -75 30	0.89	182	<0.001
Precentral_R2^b^	BA4/6	28.5 -16.5 58.5	0.66	161	<0.001
Frontal_Mid_Orb_L	BA11	-33 43.5 -18	0.61	139	<0.001
Occipital_Mid_L		-40.5 -84 -1.5	0.73	89	<0.001
Frontal_Sup_R	BA6	18 4.5 55.5	0.61	70	<0.001
Temporal_Sup_R		51 -1.5 -10.5	0.50	64	<0.001
Amygdala_R		30 -1.5 -15	0.57	59	<0.001
Precentral_L		-60 -4.5 25.5	0.50	50	<0.001
Supp_Motor_Area_L		-10.5 -3 49.5	0.61	53	<0.001
Parietal_Sup_L	BA7	-24 -66 57	0.68	43	<0.001
Frontal_Mid_L	BA8	-45 13.5 42	0.71	41	<0.001

^a^The difference in *R* value between the two groups at the peak MNI coordinate (stroke group minus healthy controls). ^b^Precentral_R1 is inferior to the seed region, and Precentral_R2 is superior to the seed region.

**Table 4:** Associations of regional GM volume with motor function in left-sided stroke patients.

Model	Independent variable	FMA-UE			
		*R* ^2^ ^a^	*p* value^b^	Beta (SE)^c^	*p* value^d^
1	Age, gender	0.148	0.124	—	—
1A	Model 1+stroke type	0.166	0.202	6.481 (8.996)	0.477
1B	Model 1a+lesion size^e^	0.311	0.54	-46.715 (20.725)	0.03
1C	Model 1b+duration^f^	0.322	0.091	-0.897 (1.483)	0.551
2	Model 1c+TIV^g^	0.430	0.037	0.093 (0.046)	0.054
3A	Model 2+ipsilesional M1^h^	0.657	<0.001	270.253 (73.767)	0.001^i^
3B	Model 2+Precentral_R	0.586	0.004	225.649 (82.359)	0.012^i^
3C	Model 2+Frontal_Inf_Oper_R	0.584	0.005	138.297 (51.101)	0.013^i^
3D	Model 2+Cerebelum_Crus1_R	0.572	0.006	121.830 (47.561)	0.018^i^
3E	Model 2+Cerebelum_Crus2_L	0.572	0.006	146.721 (57.527)	0.018^i^
3F	Model 2+Thalamus_R	0.569	0.006	364.958 (144.33)	0.019^i^
3G	Model 2+Calcarine_L	0.559	0.008	177.964 (74.344)	0.026^i^
3H	Model 2+Cuneus_L	0.541	0.011	118.519 (54.885)	0.042^i^
3I	Model 2+Precentral_L	0.536	0.012	165.151 (78.755)	0.048^i^
3J	Model 2+Precuneus_R	0.523	0.016	140.414 (72.573)	0.066
3K	Model 2+Angular_R	0.519	0.017	134.555 (71.613)	0.074
3L	Model 2+Occipital_Mid_R	0.512	0.019	130.010 (73.033)	0.089
3M	Model 2+Frontal_Inf_Orb_R	0.506	0.021	212.599 (125.31)	0.104
3N	Model 2+Frontal_Inf_Tri_L	0.503	0.022	76.607 (46.165)	0.111
3O	Model 2+Insula_R	0.485	0.031	146.124 (105.81)	0.181
3P	Model 2+Frontal_Sup_Orb_L	0.483	0.031	138.687 (102.11)	0.188
3Q	Model 2+Frontal_Sup_Orb_R	0.481	0.032	121.292 (92.458)	0.203
3R	Model 2+Rolandic_Oper_L	0.473	0.036	66.323 (56.239)	0.251
3S	Model 2+Insula_L	0.452	0.051	49.609 (67.444)	0.471

**Table 5:** Associations of regional GM volume with motor function in right-sided stroke patients.

Model	Independent variable	FMA-UE			
		*R* ^2^ ^a^	*p* value^b^	Beta (SE)^c^	*p* value^d^
1	Age, gender	0.080	0.339	—	—
1A	Model 1+TYPE	0.226	0.088	18.200 (8.364)	0.038
1B	Model 1a+lesion size^e^	0.236	0.151	12.492 (22.172)	0.578
1C	Model 1b+duration^f^	0.378	0.041	4.065 (1.779)	0.032
2	Model 1c+TIV^g^	0.413	0.048	43.456 (37.143)	0.262
3A	Model 2+Parietal_Sup_L	0.639	0.001	275.002 (66.641)	0.001^i^
3B	Model 2+Cuneus_L+R	0.572	0.006	368.137 (110.96)	0.003^i^
3C	Model 2+Amygdala_R	0.571	0.006	239.556 (72.454)	0.003^i^
3D	Model 2+Supp_Motor_Area_L	0.568	0.007	249.596 (76.286)	0.004^i^
3E	Model 2+Precentral_R2	0.567	0.006	268.241 (82.051)	0.003^i^
3F	Model 2+Occipital_Mid_L	0.565	0.007	244.802 (75.339)	0.003^i^
3G	Model 2+Frontal_Med_Orb_L	0.546	0.010	237.465 (78.268)	0.006^i^
3H	Model 2+ipsilesional M1^h^	0.535	0.013	306.615 (101.34)	0.006^i^
3I	Model 2+Occipital_Sup_R	0.528	0.014	221.261 (77.808)	0.009^i^
3J	Model 2+Precentral_R1	0.526	0.015	241.610 (85.909)	0.010^i^
3K	Model 2+Frontal_Sup_Medial_L	0.519	0.017	290.159 (105.56)	0.012^i^
3L	Model 2+Precentral_L	0.508	0.021	188.201 (71.887)	0.016^i^
3M	Model 2+Temporal_Sup_R	0.499	0.024	219.083 (86.571)	0.019^i^
3N	Model 2+Rolandic_Oper_L	0.496	0.025	251.534 (100.76)	0.021^i^
3O	Model 2+Thalamus_L	0.480	0.033	221.416 (95.529)	0.031^i^
3P	Model 2+Temporal_Inf_L	0.351	0.184	76.921 (100.115)	0.451
3Q	Model 2+Frontal_Sup_R	0.368	0.152	73.967 (89.286)	0.417
3R	Model 2+Frontal_Mid_L	0.430	0.069	209.675 (119.87)	0.095

The table shows the results of the multivariate regression model. The variables were added to the regression model in a stepwise fashion. ^a^*R*^2^ represents the goodness-of-fit of the regression model; ^b^the significance level of the regression model; ^c^unstandardized coefficient (beta) with SE (standard error) of the explained variable; ^d^the significance level of the regression coefficient of the explained variable; ^e^the lesion size was normalized by TIV (% of TIV); ^f^duration: disease duration; ^g^TIV: total intracranial volume; ^h^ipsilesional M1: the seed region; ^i^the significant variables in the regression model and regression coefficient.

## Data Availability

The data generated for this study are available from the corresponding author on reasonable request.
